# Alpha-tocopherol exerts protective function against the mucotoxicity of particulate matter in amphibian and human goblet cells

**DOI:** 10.1038/s41598-020-63085-6

**Published:** 2020-04-10

**Authors:** Hee-Sun Yang, Hyo Jung Sim, Hanna Cho, Woo Young Bang, Ha Eun Kim, Taeg Kyu Kwon, Taejoon Kwon, Tae Joo Park

**Affiliations:** 1 Biological and Genetic Resources Assessment Division, National Institute of Biological Resources, Incheon, 22689 Korea; 2 School of Life Sciences, Ulsan National Institute of Science and Technology (UNIST), Ulsan, 44919 South Korea; 3 Department of Immunology, School of Medicine, Keimyung University, Daegu, South Korea; 4 Center for Genomic Integrity, Institute for Basic Science, Ulsan, 44919 Republic of Korea

**Keywords:** Biological models, Mechanisms of disease, Natural hazards

## Abstract

Exposure to particulate matter (PM) in ambient air is known to increase the risk of cardiovascular disorders and mortality. The cytotoxicity of PM is mainly due to the abnormal increase of reactive oxygen species (ROS), which damage cellular components such as DNA, RNA, and proteins. The correlation between PM exposure and human disorders, including mortality, is based on long-term exposure. In this study we have investigated acute responses of mucus-secreting goblet cells upon exposure to PM derived from a heavy diesel engine. To this end, we employed the mucociliary epithelium of amphibian embryos and human Calu-3 cells to examine PM mucotoxicity. Our data suggest that acute exposure to PM significantly impairs mucus secretion and results in the accumulation of mucus vesicles in the cytoplasm of goblet cells. RNA-seq analysis revealed that acute responses to PM exposure significantly altered gene expression patterns; however, known regulators of mucus production and the secretory pathway were not significantly altered. Interestingly, pretreatment with α-tocopherol nearly recovered the hyposecretion of mucus from both amphibian and human goblet cells. We believe this study demonstrates the mucotoxicity of PM and the protective function of α-tocopherol on mucotoxicity caused by acute PM exposure from heavy diesel engines.

## Introduction

Recent research has highlighted that particulate matter (PM) in ambient air is globally associated with increased mortality due to cardiovascular and pulmonary disorders^1–3^. The composition of PM varies depending on the location and source of pollutants, but the major components are polycyclic aromatic hydrocarbons (PAH) and transition metals^4^, which are known to damage cellular components. The cytotoxicity of PM in ambient air is widely studied and has been specifically linked to oxidative stress induced by reactive oxygen species (ROS)^5^. The major target of oxidative damage is DNA, which causes mutagenesis and increases the risk of cardiovascular disorders and the incidence of lung cancer^6,7^. The size of PM varies from 1 nm to 100 μm. Particularly, PM2.5, which is smaller than 2.5 μm, penetrates the mucus barrier on top of the mucociliary epithelium and directly affects the cardiovascular system and pulmonary tissues^8–10^.

The mucociliary epithelium in the human airway tract protects vulnerable internal pulmonary systems from pollutants and infectious microbes in ambient air. The mucociliary epithelium is composed of two major cell types: mucus-secreting goblet cells and multi-ciliated cells. The goblet cells produce mucus vesicles that are secreted to form a mucus barrier on top of the mucociliary epithelium. It is known that two regulatory mechanisms control the secretion of mucus vesicles: baseline secretion maintains mucus barrier homeostasis, and the stimulatory pathway regulates mucus secretion in response to external stimuli^11,12^. The mucus vesicles contain gel-forming mucin proteins such as Muc5AC and Muc5B. The motile cilia on top of the multi-ciliated cells generate the directional flow of the mucus and expectorate the mucoid fluid^13^. Imbalance in mucus secretion causes respiratory diseases such as asthma, chronic obstructive pulmonary disease (COPD), and cystic fibrosis^14^.

The embryonic epithelium of amphibians is composed of mucus-secreting goblet cells and multi-ciliated cells which protect the animal from environmental challenges such as infectious microbes and contaminants^15–18^. Similarities between the embryonic mucociliary epithelium of amphibians and the human airway epithelium enables relevant *in vivo* approaches which are not feasible using other experimental models such as mice or immortalized cell lines. Amphibian embryos can be easily obtained because a single female can be induced to ovulate by injecting human chorionic gonadotrophin (HCG), after which it will lay several hundred eggs. *In vitro* fertilization synchronizes the developmental process, and the mucociliary epithelium develops within 2 days after fertilization. Additionally, the mucociliary epithelium is exposed to the outer skin, making it an experimental model of choice. Previously, we have shown that the embryonic epithelium of *Xenopus laevis* is an alternative *in vivo* model to study the pathophysiology of mucociliary epithelium and perform high-throughput drug screening for muco-active reagents^16^.

In this study, we have examined the acute toxicity of PM from a heavy diesel engine to the mucociliary epithelium using amphibian embryos and human goblet cells. Our data demonstrate that a reduction in mucus secretion from goblet cells is a conserved and acute response to PM exposure, the response of which may be relieved by α-tocopherol.

## Results

### The mucociliary epithelium of *Bombina orientalis* is favorable for detecting acute mucus secretion response to exogenous stimuli

Previous studies demonstrated that exposure to PM results in DNA damage by increasing ROS. In addition, long-term exposure to PM was shown to damage cardiovascular systems, respiratory tracts, and increase the risk of cancer and mortality^6,19–22^. However, the response of the respiratory tract to acute PM exposure is not fully understood. A recent study suggests that transcriptional responses to acute PM exposure significantly change the gene expression profiles of human bronchial epithelial cell lines^23^. This suggests that the defensive function of the mucociliary epithelium may be compromised by acute PM exposure before the accumulation of oxidative damage by ROS and associated consequences of long-term PM exposure. However, the current research model is not an appropriate system to examine acute responses of mucociliary epithelium due to its limited availability and the complexity of analysis tools.

**Figure 1:**
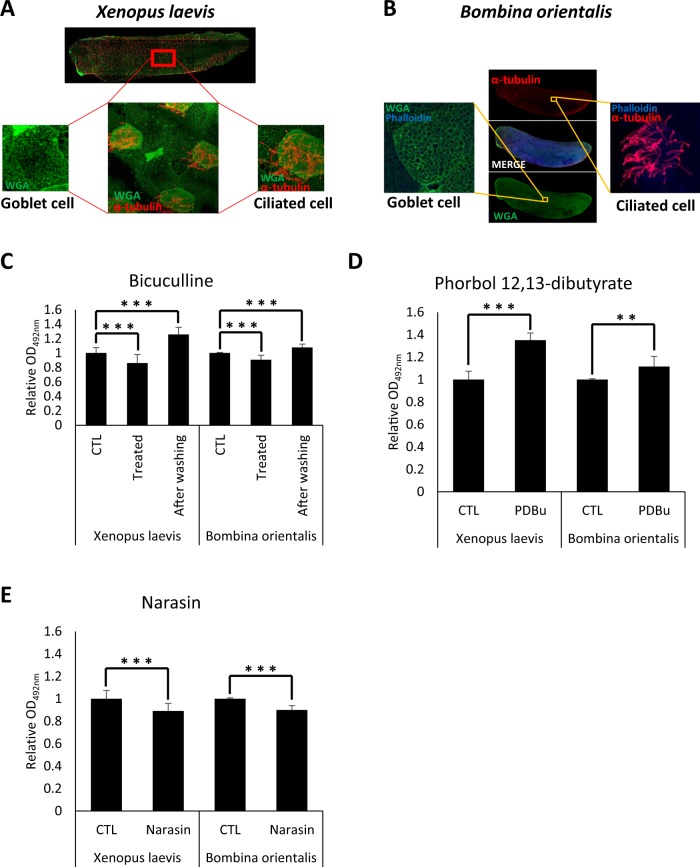
The mucociliary epithelium of amphibian embryos is structurally and physiologically similar to human airway epithelium. (**A**) The embryonic epithelium of *Xenopus laevis* was visualized by fluorescent imaging analysis. Goblet cells were stained with WGA-Alexa 488 and multi-cilia were stained with anti-acetylated tubulin antibody. (**B**) The embryonic epithelium of *Bombina orientalis* was visualized by the same protocol. (**C**) Bicuculline reversibly inhibited mucus secretion from the goblet cells of *X. laevis* and *B. orientalis* embryonic epithelium. Statistical analysis was performed using one-way ANOVA. From left to right, n = 57, 42, 39, 17, 5, 8. (**D**) Phorbol 12,13-dibutyrate increased mucus secretion from the goblet cells of *X. laevis* and *B. orientalis* embryonic epithelium. Statistical analysis was performed using Student’s t-test. From left to right, n = 57, 42, 17, 18. (**E**) Narasin inhibited mucus secretion from *X. laevis* and *B. orientalis* embryonic epithelium. Statistical analysis was performed using Student’s t-test. From left to right, n = 57, 42, 17, 18. Asterisks represent: ***(p < 0.001), **(0.001 < p < 0.01), *(0.01 < p < 0.05), ns (0.05 < p).

In a previous study, we developed an alternative research model for studying mucus secretion and successfully identified potential muco-active reagents using the embryonic mucociliary epithelium of the amphibian *X. laevis*^16^. To evaluate the effect of PM in the environment, we used another amphibian embryo model to analyze the acute response of mucociliary epithelium upon exposure to PM from a heavy diesel engine. Like *X. laevis*, the embryonic epithelium of *Bombina orientalis* was examined and found to have mucus-secreting goblet and multi-ciliated cells very similar to the human mucociliary epithelium in the airway tract (Fig. 1A,B). We further examined if the mucociliary epithelium is physiologically similar to that of human airway epithelium by treating with known muco-active reagents. The mucus secretion level was measured by WGA-HRP (HRP-conjugate wheat germ agglutinin) as previously described^16^.

Known muco-regulators such as bicuculline and phorbol 12,13-dibutyrate significantly upregulated mucus secretion from the embryonic epithelium of *B. orientalis* (Fig. 1C,D). Narasin also significantly reduced mucus secretion as previously shown (Fig. 1E)^16^. These data suggest that the embryonic epithelium of *B. orientalis* offers similar utility as *X. laevis* for studying the physiology of mucociliary epithelium. We have also found that *B. orientalis* embryos are more favorable for embryonic manipulation due to their large size compared to *X. laevis* embryos.

### PM decreases mucus secretion from goblet cells in the mucociliary epithelium of *B. orientalis*

Next, we performed a series of experiments to investigate the acute response of mucus secretion to PM exposure. To examine the effects of PM on mucociliary epithelium, we treated *B. orientalis* embryos with increasing doses of PM from a heavy diesel engine. Secreted mucus from embryonic mucociliary epithelium 3 h after PM treatment in nursing media was adhered to a multi-well plate, and adhered mucus levels were measured by enzyme-linked lectin assay (ELLA) using WGA-HRP, as previously described^16^.

**Figure 2:**
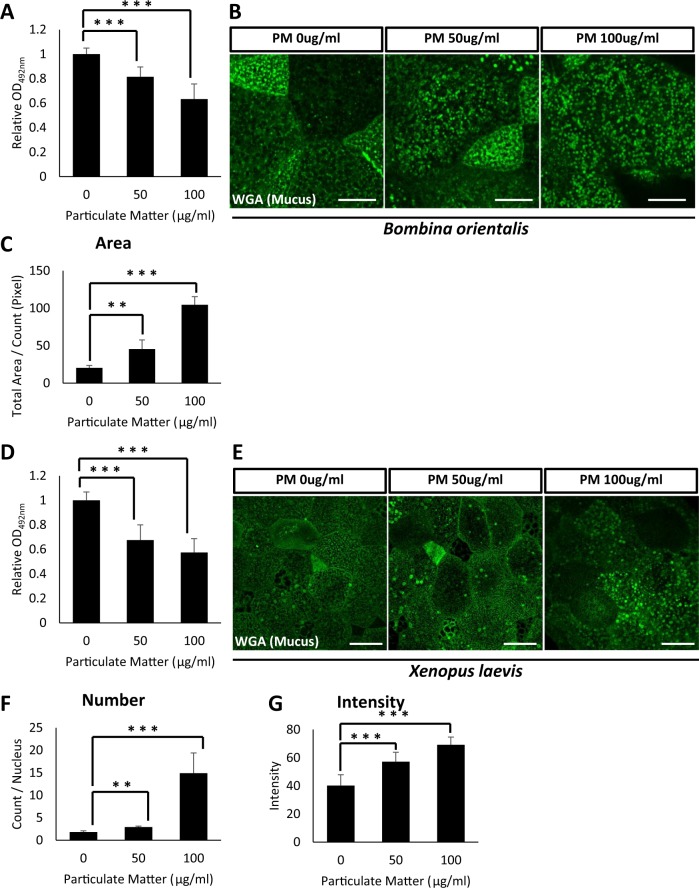
Particulate matter from heavy diesel engine disturbs mucus secretion from the mucociliary epithelium in *B. orientalis* and *X. laevis* embryos. (**A**) Increasing doses of PM were administered to *B. orientalis* embryos and secreted mucus levels were analyzed by ELLA using WGA-HRP. 50 µg/mL or 100 µg/mL of PM significantly disturbed mucus secretion from the embryonic epidermis. Statistical analysis was performed using one-way ANOVA. From left to right, n = 21, 16, 20. (**B**) The WGA positive mucus vesicles were visualized by fluorescent microscopy. PM exposure significantly accumulated mucus vesicles in the cytoplasm. Scale bar = 10 μm. (**C**) Quantification of WGA positive mucus vesicles in (**B**). Statistical analysis was performed using one-way ANOVA. From left to right, n = 4, 4, 4. (**D**) Particulate matter-induced hyposecretion of mucus was recovered by α-tocopherol in *Xenopus* embryos. Statistical analysis was performed using one-way ANOVA. From left to right, n = 24, 22, 22. (**E**) The WGA positive mucus vesicles were visualized by fluorescent microscopy. The exposure to the particulate matter significantly accumulated the mucus vesicles in the cytosol. Scale bar = 20 μm. (**F,G**) Quantification of WGA positive mucus vesicles in (**E**). For (**F**), statistical analysis was performed using one-way ANOVA. From left to right, n = 4, 4, 4. For (**G**), statistical analysis was performed using one-way ANOVA. From left to right, n = 30, 30, 30. Asterisks represent ***(p < 0.001), **(0.001 < p < 0.01), *(0.01 < p < 0.05), ns (0.05 < p). PM, particulate matter; ELLA, enzyme-linked lectin assay.

ELLA results indicated that increasing doses of PM significantly reduced secreted mucus levels from embryonic mucociliary epithelium rather than increasing mucus secretion (Fig. 2A). We further examined whether this reduction in mucus secretion was due to the cytoplasmic accumulation of mucus vesicles by staining with WGA-Alexa 488. Indeed, PM-treated embryos retained an increased number of mucus vesicles in their cytoplasm compared to those of the control (Fig. 2B,C).

We next confirmed the mucotoxicity of PM using *X. laevis* embryos. Consistent with the *B. orientalis* data, PM treatment significantly decreased mucus secretion. (Fig. 2D). The reduction in mucus secretion was mainly due to the defective secretion of mucus vesicles as was seen in *B. orientalis* embryos, in which WGA-labeled mucus vesicles accumulated in the cytoplasm of goblet cells that significantly worsened with increasing doses of PM (Fig. 2E–G).

### The impact of acute PM exposure on mucus secretion-related gene expression

**Figure 3:**
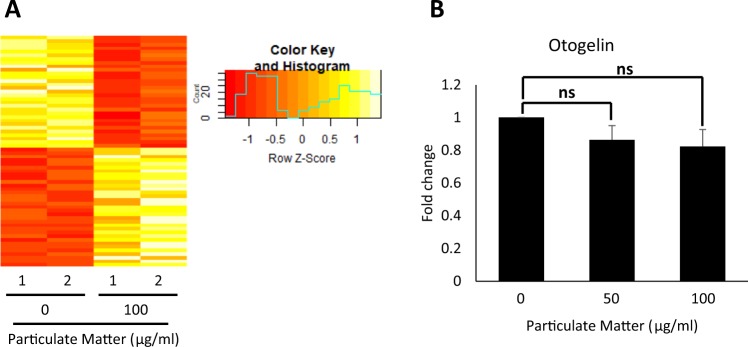
Acute exposure to particulate matter changes gene expression, but did not significant alter expression of mucus-related genes.(**A**) RNA-seq and differentially expressed gene (DEG) analysis revealed that PM exposure significantly changed the gene expression profile of *X. laevis* embryos. (**B**) Otogelin expression was slightly reduced by PM treatment without statistical significance. Statistical analysis was performed using Student’s t-test. From left to right, n = 3, 3, 3. Asterisks represent ***(p < 0.001), **(0.001 < p < 0.01), *(0.01 < p < 0.05), ns (0.05 < p). PM, particulate matter.

We examined the molecular mechanism for PM mucotoxicity in mucociliary epithelium by analyzing changes in gene expression profiles upon PM treatment. RNA-seq and gene enrichment analysis for differentially expressed genes (DEG) after PM treatment revealed that the transcriptome of PM-exposed embryos was significantly changed compared to control embryos (Fig. 3A, Supplementary Table S1). However, GO term pathway analysis on the DEG revealed that mucus-related genes were not significantly affected by the acute PM exposure (Supplementary Table S2). We did confirm that the expression of otogelin, a known mucus component, was slightly reduced by PM treatment, although without statistical significance (Fig. 3B). These data suggest that the mucociliary epithelium is a vulnerable target of acute PM exposure; however, changes to gene expression involving mucus secretion or production were not the major cause of defective mucus secretion induced by exposure to PM.

### α-tocopherol protects against PM-induced hyposecretion of mucus in *X. laevis* and *B. orientalis*

**Figure 4:**
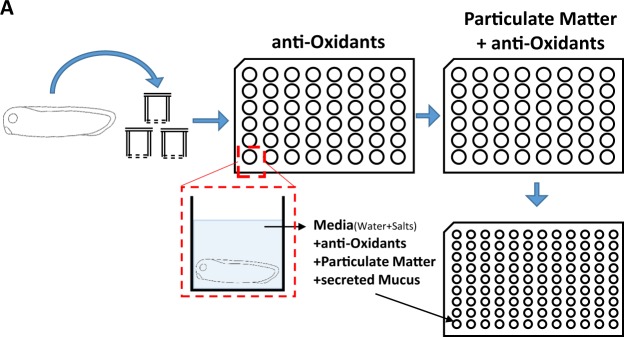
Schematic of experimental procedure to analyze protective functions of antioxidants on particulate matter mucotoxicity. *X. laevis* embryos were pretreated with the indicated antioxidants trolox, NAC (N-acetyl cysteine), or α-tocopherol. After 1 h incubation in the antioxidant-containing media, embryos were transferred to PM treatment media. The level of secreted mucus was measured by ELLA assay using WGA-HRP. PM, particulate matter; ELLA, enzyme-linked lectin assay.

**Figure 5:**
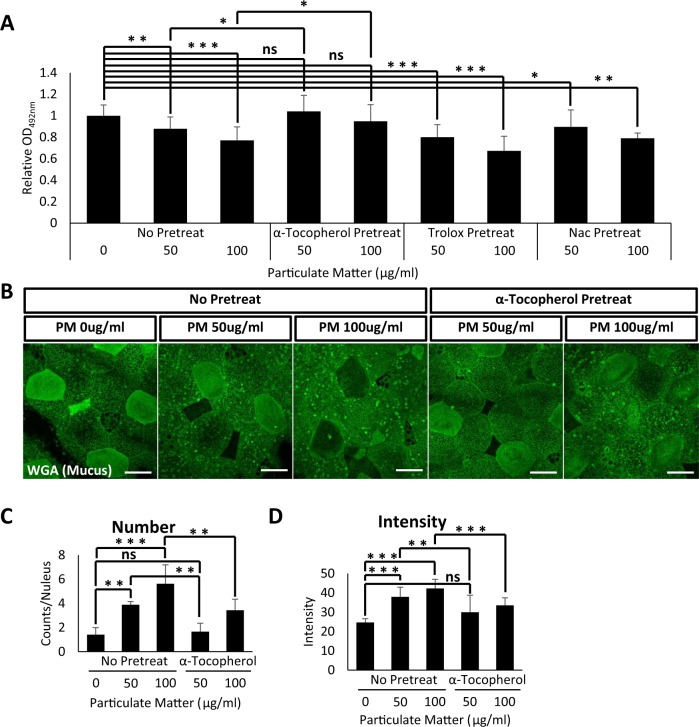
α-tocopherol treatment protected *X. laevis* mucociliary epithelium from particulate matter mucotoxicity. (**A**) Indicated doses of antioxidants administered to *X. laevis* embryos followed by PM. Secreted mucus from *X. laevis* embryonic mucociliary epithelium was measured by ELLA using WGA-HRP. Pretreatment with antioxidants trolox and NAC (N-acetyl cysteine) did not affect PM-induced mucus hyposecretion. However, pretreatment with α-tocopherol significantly recovered the reduction in mucus secretion induced by PM. Statistical analysis was performed using one-way ANOVA. From left to right, n = 52, 14, 12, 7, 6, 7, 7, 6, 4. (**B**) WGA positive mucus vesicles accumulated in embryonic mucociliary epithelium were visualized by fluorescent microscopy. Scale bar = 20 μm. (**C,D**) Quantification of WGA positive mucus vesicles in (**B**). Statistical analysis was performed using one-way ANOVA. For (**C**), from left to right, n = 6, 4, 6, 4, 6. For (**D**), from left to right, n = 12, 13, 14, 11, 14. Pretreatment with α-tocopherol ameliorated the accumulation of mucus vesicles induced by PM. Asterisks represent ***(p < 0.001), **(0.001 < p < 0.01), *(0.01 < p < 0.05), ns (0.05 < p). PM, particulate matter; ELLA, enzyme-linked lectin assay.

The major cause of cytotoxicity upon PM exposure is caused by excessive ROS. We therefore reasoned that antioxidant treatment may recover the reduced mucus secretion in PM-matter treated embryonic epithelium. For this experiment, we used three well-known antioxidants: Trolox, NAC (N-acetyl cysteine), and α-tocopherol. To this end, we pretreated *X. laevis* embryos with various doses of antioxidants for 1 h, followed by 3 h of PM treatment. Mucus secretions were then compared to those of PM-treated embryos without antioxidant pretreatment (Fig. 4). To our surprise, trolox and NAC had minimal effects on mucus secretion recovery; however, α-tocopherol-treated embryos displayed mucus recovery to normal levels of secretion (Fig. 5A).

**Figure 6:**
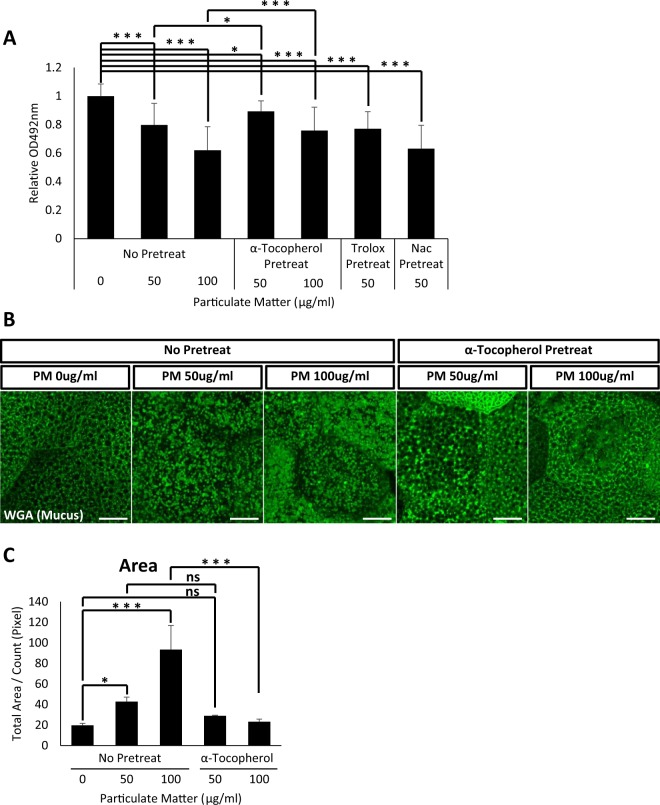
α-tocopherol treatment protects *B. orientalis* mucociliary epithelium from PM mucotoxicity. (**A**) Indicated doses of antioxidants were administered to *B. orientalis* embryos followed by PM exposure, and secreted mucus from *B. orientalis* embryonic mucociliary epithelium was measured by ELLA using WGA-HRP. Pretreatment with antioxidants trolox and NAC (N-acetyl cysteine) did not affect PM-induced mucus hyposecretion. However, pretreatment with α-tocopherol significantly recovered the reduction in mucus secretion induced by PM. Statistical analysis was performed using one-way ANOVA. From left to right, n = 36, 32, 36, 34, 31, 36. (**B**) WGA positive mucus vesicles accumulated in embryonic mucociliary epithelium were visualized by fluorescent microscopy. Scale bar = 10 μm. (**C**) Quantification of WGA positive mucus vesicles in (**B**). Pretreatment with α-tocopherol ameliorated the accumulation of mucus vesicles induced by PM. Statistical analysis was performed using one-way ANOVA. From left to right, n = 7, 6, 7, 4, 6. Asterisks represent ***(p < 0.001), **(0.001 < p < 0.01), *(0.01 < p < 0.05), ns (0.05 < p). PM, particulate matter; ELLA, enzyme-linked lectin assay.

Next, we examined if α-tocopherol induces mucus secretion recovery by facilitating mucus vesicle secretion. Consistent with previous data, mucus vesicles accumulated in the cytoplasm upon treatment with PM. However, pretreatment with α-tocopherol prior to PM exposure significantly reduced the accumulation of mucus vesicles (Fig. 5B–D). We also examined the protective effects of α-tocopherol using *B. orientalis* embryos and obtained similar results (Fig. 6A–C). These data indicate that reductions in mucus secretion may be a common cytotoxic outcome of PM exposure for mucus-secreting goblet cells. Also, our data suggest that α-tocopherol protects mucociliary epithelium from PM mucotoxicity.

### ROS are not the major cause of PM-induced mucus hyposecretion

We speculated that increased ROS may be a direct cause of mucus hyposecretion, and pretreatment with α-tocopherol could recover secretion by reducing ROS levels. To this end, we measured ROS levels induced by PM treatment. Consistent with previous reports, PM increased cellular ROS levels measured by H_2_DCFDA (Supplementary Fig. 1). We then pretreated *X. laevis* embryos with the antioxidants trolox, NAC, and α-tocopherol for 1 h, followed by 3 h of PM treatment. To our surprise, among the antioxidants, trolox pretreatment effectively recovered PM-induced ROS to normal levels, while α-tocopherol did not affect ROS (Supplementary Fig. 1). Next, we measured other known ROS-dependent cytotoxicities of PM such as cell death and DNA damage by performing TUNEL and γ-H2AX immunostaining assays, respectively. However, acute PM exposure did not significantly cause cell death or DNA damage in our *in vivo X. laevis* model (Supplementary Fig. 2). These data indicate that PM-induced ROS are not directly affecting mucus hyposecretion, *per se*. Rather, there are unknown components in PM that may cause hyposecretion of mucus from the mucociliary epithelium.

### α-tocopherol protects against PM-induced mucus hyposecretion in Calu-3 human goblet cells

**Figure 7:**
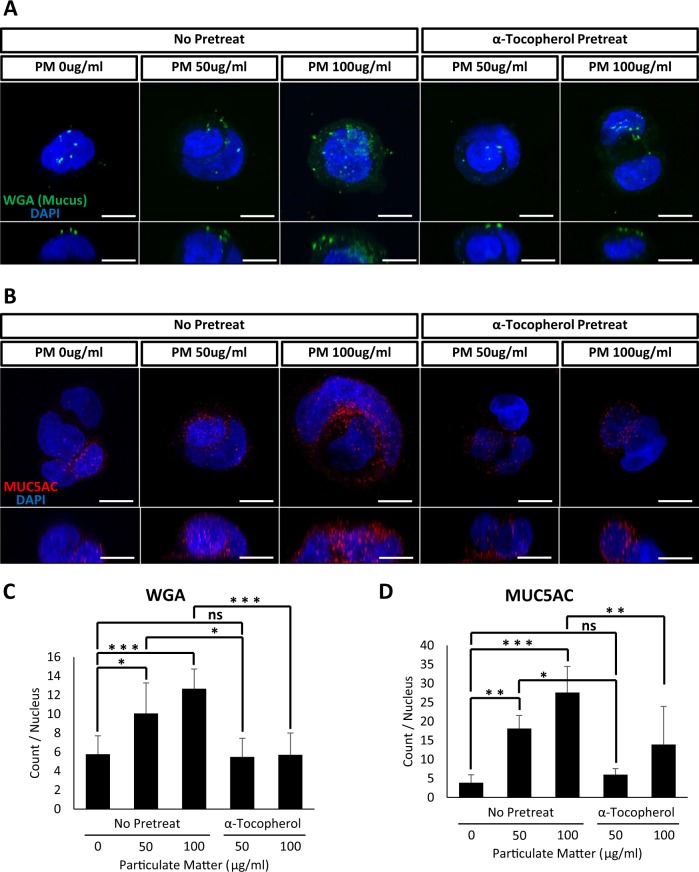
Particulate matter induced the accumulation of mucus vesicles in human Calu-3 goblet cells. (**A**) PM treatment induced the accumulation of WGA positive mucus vesicles in Calu-3 cells. Mucus vesicles were stained with WGA-Alexa 488. Scale bar = 10 μm. (**B**) PM treatment induced the accumulation of MUC5AC positive mucus vesicles in Calu-3 cells. MUC5AC vesicles were stained with an anti-MUC5AC antibody. Consistent with the amphibian mucociliary epithelium, pretreatment with α-tocopherol significantly reduced the accumulation of mucus vesicles induced by PM in Calu-3 cells. Scale bar = 10 μm. (**C,D**) Quantification of WGA and MUC5AC positive vesicles from (**A**) and (**B**), respectively. Statistical analysis was performed using one-way ANOVA. For (**C**), from left to right, n = 6, 4, 6, 8, 8. For (**D**), n = 6, 5, 7, 4, 5. Asterisks represent ***(p < 0.001), **(0.001 < p < 0.01), *(0.01 < p < 0.05), ns (0.05 < p). PM, particulate matter.

We next examined the mucotoxic effects of PM in human cells. Calu-3 cells have been frequently used to study the pathophysiology of goblet cells^24^. We treated Calu-3 cells with PM and analyzed the accumulation of cytoplasmic mucus vesicles using WGA-Alexa 488. Consistent with data obtained using embryonic mucociliary epithelium in *B. orientalis* and *X. laevis*, acute PM exposure significantly increased cytoplasmic mucus granules (Fig. 7A). We also confirmed that the accumulated cytoplasmic vesicles contained mucin by performing fluorescent immunostaining using an anti-MUC5AC antibody (Fig. 7B). Indeed, MUC5AC-positive mucus vesicles were significantly enriched in PM-treated Calu-3 cells (Fig. 7B,D). Furthermore, pretreating with α-tocopherol rescued the abnormal accumulation of mucus granules, which were examined by either WGA-Alexa 488 or anti-MUC5AC staining (Fig. 7A–D). These data strongly suggest that the abnormal accumulation of mucus granules in goblet cells is a common response to PM exposure, which reduces the level of secreted mucus from the mucociliary epithelium including human airway epithelium.

## Discussion

PM in the ambient air is known to contain biologically toxic compounds such as PAH and transition metals. These toxic compounds in PM penetrate mucus barriers in the mucociliary epithelium of the human airway tract and directly affect human health. The major cause of cytotoxicity from PM is a known increase in ROS. Abnormal increases in ROS damage cellular components such as DNA and proteins, thereby causing mutations to accumulate. This cytotoxicity consequently increases the risk of cardiovascular and pulmonary disorders. The correlation between PM exposure and human disorders is based on long-term exposure. However, recent studies also suggest that the acute response to PM also causes significant changes in gene expression and the physiology of live organisms.

The mucociliary epithelium in the human airway tract is an essential tissue that functions as a defense mechanism against environmental challenges such as pathogens and toxic pollutants. Therefore, most respiratory disorders, such as COPD, asthma, and respiratory infections, often disrupt normal airway mucociliary function. In this study, we examined the acute response of the mucociliary epithelium to PM exposure from heavy diesel engines. To this end, we utilized amphibious embryonic skin models. Our data suggest that acute exposure to PM significantly impairs mucus secretion and results in the accumulation of mucus vesicles within the cytoplasm of goblet cells. In addition, mucus hyposecretion from the mucociliary epithelium upon PM exposure may be a consequence of disrupting the mucus vesicle secretory pathway. Consistent with this idea, RNA-seq analysis demonstrated that the expression of genes involved in mucus production pathways were not significantly affected by PM exposure.

Interestingly, pretreatment of α-tocopherol elicited a near complete recovery of mucus hyposecretion from the mucociliary epithelium. Furthermore, consistent with the amphibian data, pretreatment of α-tocopherol completely reversed PM-induced mucus accumulation in human Calu-3 cells. These data imply that PM may cause very common toxicity across mucus-secreting goblet cells, and this mucotoxicity is an immediate response rather than a consequence of long-term exposure to PM.

Although the major cytotoxic components of PM are PAH and transition metals, PM has very diverse composition depending on many environmental factors such as cities, seasons, and air pollutants. Therefore, one limitation of this study is that the composition of PM, exhaust from heavy diesel engines (purchased from Sigma-Aldrich), is not an exact match to naturally occurring PM without a defined chemical composition. For this reason, we were unable to identify the component responsible for mucotoxicity on the mucus-secreting cells. Further research needs to be performed to identify the most important mucotoxic components of PM.

**Figure 8:**
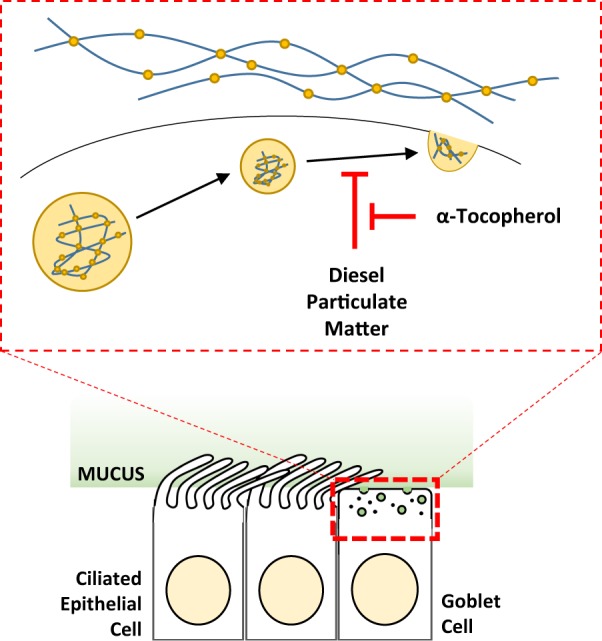
Schematic of the protective effect of α-tocopherol on particulate matter mucotoxicity. Our data suggest that acute exposure to PM causes mucus hyposecretion, likely through interference with secretory and exocytic processes in goblet cells. α-tocopherol offers a protective function over PM mucotoxicity. PM, particulate matter.

Altogether, we believe this study clearly demonstrates that mucus hyposecretion is a conserved and common response of mucociliary epithelium to PM exposure. Furthermore, our data present clear evidence that α-tocopherol has a protective capacity against the mucotoxicity of PM from heavy diesel engines (Fig. 8).

## Materials and Methods

### Embryo manipulation

*Xenopus laevis* and *Bombina orientalis* adult females were ovulated using 380 units of human chorionic gonadotropin (HCG) a day before. For *B. orientalis*, an adult male was also ovulated using 280 units of HCG a day before for natural mating. For *X. laevis* egg fertilization, a testicle was dissected from an adult male, ground, and sprayed on the squeezed *X. laevis* eggs. To remove the jelly layer, a 3% cysteine pH 7.9 solution was used for *X. laevis* embryos and a 3% cysteine pH 9.5 solution for *B. orientalis* embryos. Fertilized *X. laevis* embryos were grown in 1/3× MMR and fertilized. *B. orientalis* embryos were grown in sterilized water until used. The Institutional Animal Care and Use Committee (IACUC) of Ulsan National Institute of Science and Technology (UNIST) approved this work (Reference number, UNISTIACUC-16-14). All methods were performed in accordance with the relevant guidelines and regulations

### Enzyme-linked lectin assay (ELLA) for secreted mucus detection

Embryos were washed using sterilized 1/3× MMR buffer for *X. laevis* and sterilized water for *B. orientalis* to remove secreted mucus particles which remain on the epithelium. After washing, five embryos were incubated with each compound. Treatments consisted of bicuculline and narasin at 10 μM and phorbol 12,13-dibutyrate at 1 μM for 3 h. Antioxidant pretreatments occurred for 1 h, after which diesel PM treatment was carried out for 3 h. Media was collected as a time series into a U-shaped bottom 96-well plate. Collected media was incubated overnight with sodium azide at 4 °C. The next day, the incubated 96-well plates were washed twice using PTW (1 × PBS, 0.1% Tween-20), then blocked using 1% BSA for 1 h. After blocking, HRP-conjugated wheat germ agglutinin (WGA) was diluted to 1/10,000 in 1% BSA and also incubated for 1 h. After incubation, the 96-well plates were washed twice using PTW. For chromogenic detection, one tablet of o-phenylenediamine dihydrochloride (SIGMA P8289) was dissolved in 30 mL phosphate citrate buffer (0.1 M dibasic sodium phosphate, 0.05 M sodium citrate, pH 5.0) and 3 μL hydrogen peroxide was added before use. The reaction was stopped using 2.5 N sulfuric acid. Optical density was detected by a microplate reader (molecular devices, Spectramax 190) using 492-nm wavelength. All solutions used in this step were sterilized.

### Cell culture

Calu-3, which is a human lung epithelial cell line (ATCC HRB-55), was cultured using DMEM (Gibco 11995) in 5% carbon dioxide. Cells were harvested using trypsin-EDTA (Gibco, 25200) and spun down at 1,300 rpm for 5 min. For diesel PM treatments, 4 × 10^5^ cells were cultured on a coverslip for fixation and immunofluorescence analysis.

### Immunofluorescence microscopy

Compound-treated embryos were fixed using MEMFA (1 × MEM salt, 7.5% formaldehyde) solution for 2 h at room temperature or overnight at 4 °C. Diesel PM-treated Calu-3 cells were fixed using 1% formaldehyde solution. Fixed samples were washed twice with TBST (1 × TBS, 0.1% Triton X-100) and then blocked using blocking solution (1 × TBS, 10% FBS, 2% DMSO) for 30 min at room temperature. After blocking, primary antibodies were diluted in blocking solution with sodium azide and incubated with the samples overnight at 4 °C. After primary antibody incubation, samples were washed using TBST several times. Secondary antibodies were diluted in blocking solution and then incubated with the samples for 1 h at room temperature. After secondary antibody incubation, the samples were washed in TBST several times. The embryos were imaged as whole mount, whereas cells were placed on glass slides using mounting solution (Invitrogen, P36930) for imaging. Immunofluorescent analysis was performed with the following antibodies: anti-MUC5AC (Abcam, ab77576), anti-acetylated tubulin (Sigma-Aldrich, T7451), anti-alpha tubulin (Abcam, ab15246), WGA-Alexa 488 (Molecular Probes, W11261), TUNEL (Roche, 11684809910), anti-phospho-histone H2A.X (Millipore, 05-646), phalloidin-Alexa 633 (Life Technologies, A22284), DAPI (Molecular Probes, D1306). Samples were imaged using a confocal microscope (Zeiss, LSM780). Images were analyzed using the Zen program (Zeiss).

### FACS analysis

Compound-treated Calu-3 cells were harvested using trypsin-EDTA solution (Gibco, 25200). Harvested cells were washed with PBS and stained using CM-H2DCFDA (Invitrogen, C6827) per manufacturer’s protocol. Experiments were performed using a BD Biosciences LSRFortessa cell analyzer. Data were analyzed using FlowJo software (BD Biosciences).

### Transcriptome analysis

Total RNA was extracted using Trizol reagent (Thermo Fisher, 15596018) according to manufacturer’s protocol. To eliminate DNA, samples were purified using PCI/Ethanol purification and lithium chloride methods. Library preparation and RNA sequencing were performed by Theragen using a TruSeq stranded mRNA sample prep kit (Illumina, 20020595) and HiSeq2500 (Illumina). Raw data were aligned to the *X. Laevis* transcripts downloaded from Xenbase (http://xenbase.org/, RRID:SCR_003280) using BWA (version 0.7.17) and differentially expressed genes (DEG) were analyzed using edgeR (version 3.22.5). DEG were sorted as greater than 2-fold change and less than 0.01 false discovery rate (FDR).

### Quantitative PCR

cDNA was synthesized using GoScript Reverse Transcriptase (Promega, A5003) according to manufacturer’s protocol. Real-time PCR was performed using PowerUp SYBR Green Master Mix (Thermo Fisher, A25742) and QuantStudio 5 Real-Time PCR System (Applied Biosystems, A28140). Primers were: GAPDH (F: 5′-GCCGTGTATGTGGTGGAATCT-3′, B: 5′-AAGTTGTCGTTGATGACCTTTGC-3′), and otogelin (F: 5′-TGATGACTCCAGCAAGGAAAGC-3′, B: 5′-GATAACCTGTGACTGAGCAGACACC-3′).

## Supplementary information

Alpha-tocopherol exerts protective function against the mucotoxicity of particulate matter in amphibian and human goblet cells.

## Data Availability

The authors declare that all relevant data are available upon request.
