# Supplementary material for: Alpha-tocopherol exerts protective function against the mucotoxicity of particulate matter in amphibian and human goblet cells
Source: Sci Rep. 2020 Apr 10;10:6224. doi: 10.1038/s41598-020-63085-6 (PMC7148342; doi:10.1038/s41598-020-63085-6)
Supplement: Supplementary file 1 — Alpha-tocopherol exerts protective function against the mucotoxicity of particulate matter in amphibian and human goblet cells. [file 41598_2020_63085_MOESM1_ESM.docx]

**Alpha-tocopherol exerts protective function against the mucotoxicity of particulate matter in amphibian and human goblet cells.**

**Hee-Sun Yang^1†^, Hyo Jung Sim^2†^, Hanna Cho^1^, Woo Young Bang^1^, Ha Eun Kim^2^, Taeg Kyu Kwon^3^, Taejoon Kwon^2*^, Tae Joo Park^2, 4*^**

^1^Biological and Genetic Resources Assessment Division, National Institute of Biological Resources, Incheon, 22689, Korea

^2^School of Life Sciences, Ulsan National Institute of Science and Technology (UNIST), Ulsan, 44919, South Korea.

^3^Department of Immunology, School of Medicine, Keimyung University, Daegu, South Korea

^4^Center for Genomic Integrity, Institute for Basic Science, Ulsan, 44919, Republic of Korea

^†^ These authors contributed equally to this work.

^*^Corresponding author:

**T-J Park, Ph.D.** Email: parktj@unist.ac.kr

**T Kwon, Ph.D.** Email: tkwon@unist.ac.kr

**Supplementary Information**

**Supplementary figures**

**
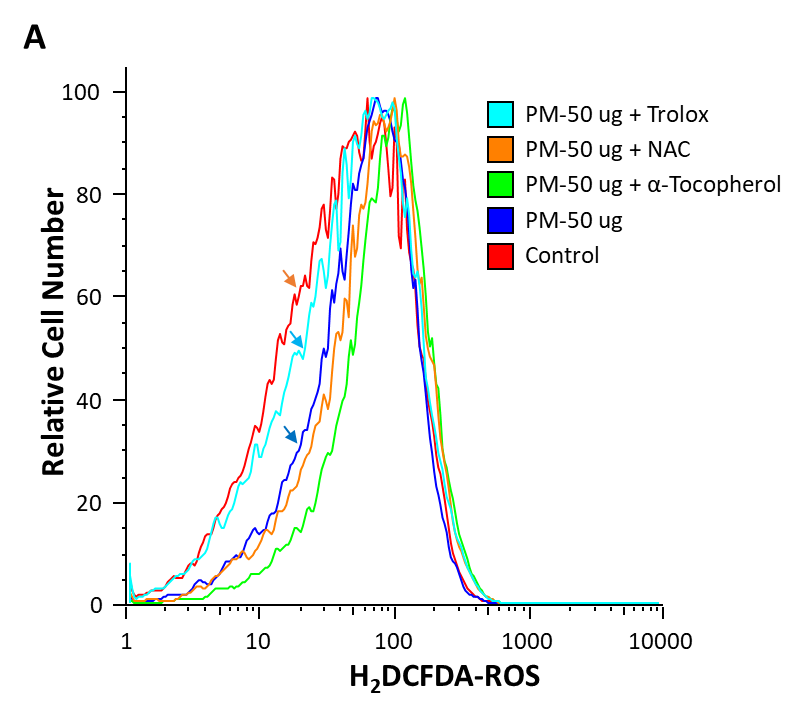
**

**Supplementary Figure 1. Particulate matter-induced ROS are not directly involved in mucus hyposecretion.**

**A.** ROS levels were measured by H_2_DCFDA-mediated FACS analysis. Trolox effectively reduced PM-induced ROS, but NAC (N-acetyl cysteine) and α-tocopherol did not. ROS, reactive oxygen species; PM, particulate matter.

**
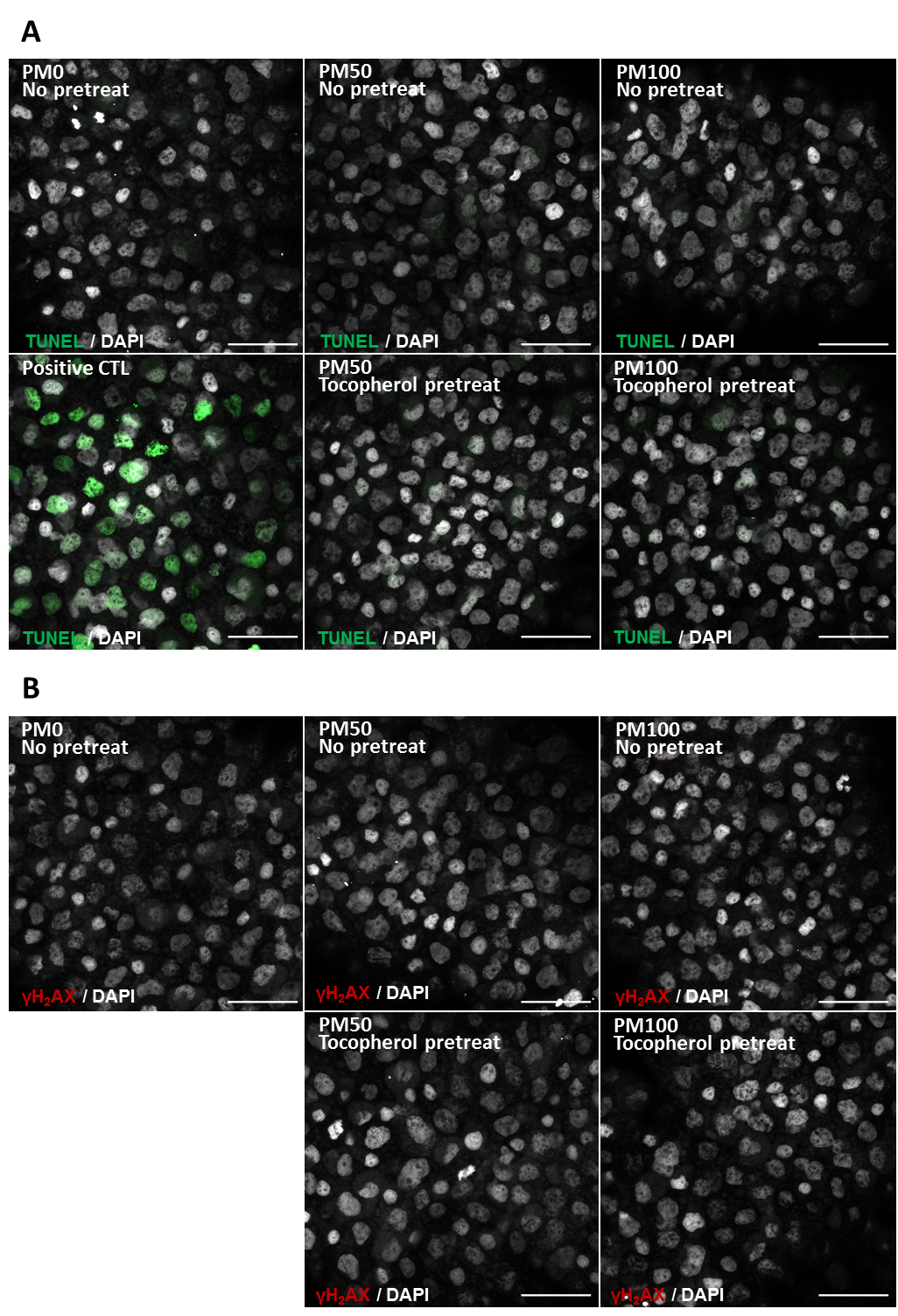
**

**Supplementary Figure 2. Acute exposure to particulate matter does not significantly affect cell death and DNA damage.**

**A.** Apoptotic cell death was visualized by TUNEL assay and fluorescent microscopy. Acute exposure to PM did not significantly induce cell death. Scale bar = 50 μm. **B.** DNA damage was visualized by γ-H2AX immunostaining. Acute exposure to PM did not significantly induce DNA damage. Scale bar = 50 μm. PM, particulate matter.

**Supplementary Table 1. The list of differentially regulated genes by particulate matter treatment**

| **Gene** | **logFC** | **logCPM** | **PValue** | **FDR** |
| --- | --- | --- | --- | --- |
| atp12a-like.1.L\|HS=ATP4A\|95\|Xelaev18026097m | 3.650081 | 3.148451 | 1.46E-52 | 3.69E-48 |
| unnamed\|HS=CDH26\|68\|Xelaev18043533m | -1.43014 | 5.225678 | 2.93E-34 | 2.47E-30 |
| unnamed\|HS=KLF2\|113\|Xelaev18001123m | -2.61056 | 2.526607 | 1.81E-29 | 1.15E-25 |
| unnamed\|HS=IRG1\|95\|Xelaev18038702m | 1.950271 | 4.11391 | 3.64E-21 | 1.53E-17 |
| LOC100494753.L\|HS=ABCB1\|98\|Xelaev18034772m | -1.2661 | 4.303996 | 7.20E-20 | 2.49E-16 |
| fos.L\|HS=FOS\|101\|Xelaev18039459m | -2.45205 | 2.930684 | 1.76E-18 | 4.95E-15 |
| unnamed\|HS=IRG1\|95\|Xelaev18038693m | 2.226972 | 3.094302 | 3.53E-15 | 8.10E-12 |
| unnamed\|HS=VENTX\|23\|Xelaev18034615m | 1.257388 | 4.058838 | 1.98E-14 | 4.17E-11 |
| unnamed\|HS=CRYM\|98\|Xelaev18047637m | 1.401065 | 3.022316 | 3.57E-14 | 6.94E-11 |
| egr1.L\|HS=EGR1\|100\|Xelaev18017353m | -2.13697 | 2.598771 | 5.86E-13 | 9.88E-10 |
| sik1.S\|HS=SIK1\|42\|Xelaev18014474m | -1.39862 | 4.176865 | 7.19E-13 | 1.14E-09 |
| fos.S\|HS=FOS\|101\|Xelaev18041369m | -2.13071 | 3.370641 | 5.25E-12 | 7.80E-09 |
| unnamed\|HS=ANGPTL5\|64\|Xelaev18021227m | 1.183536 | 3.139094 | 1.92E-11 | 2.42E-08 |
| unnamed\|HS=HBZ\|100\|Xelaev18047459m | -2.31224 | 2.000171 | 6.41E-10 | 6.00E-07 |
| unnamed\|HS=PLEKHS1\|23\|Xelaev18036639m | 1.024888 | 3.358928 | 8.48E-10 | 7.65E-07 |
| unnamed\|HS=IRG1\|18\|Xelaev18038707m | 2.329747 | 1.155036 | 9.22E-10 | 8.03E-07 |
| elf3.S\|HS=ELF3\|104\|Xelaev18014944m | 1.037326 | 3.440821 | 2.74E-09 | 1.98E-06 |
| unnamed\|HS=NKG7\|89\|Xelaev18036319m | 2.50692 | 1.108786 | 2.38E-08 | 1.37E-05 |
| dhrs7b.S\|HS=DHRS7B\|95\|Xelaev18047447m | 1.627619 | 2.013891 | 3.99E-08 | 2.05E-05 |
| fgb.L\|HS=FGB\|92\|Xelaev18005467m | -1.45564 | 2.239848 | 7.39E-08 | 3.40E-05 |
| unnamed\|HS=NA\|00\|Xelaev18036325m | 1.460858 | 4.092102 | 1.18E-07 | 4.75E-05 |
| elf3.L\|HS=ELF3\|104\|Xelaev18012239m | 1.203571 | 3.118728 | 3.18E-07 | 0.000112 |
| unnamed\|HS=PHYH\|26\|Xelaev18038704m | 1.157465 | 2.976352 | 1.32E-06 | 0.000389 |
| unnamed\|HS=ZNF160\|66\|Xelaev18030394m | -1.68121 | 1.132386 | 1.94E-06 | 0.000544 |
| unnamed\|HS=GPLD1\|11\|Xelaev18031727m | 2.801476 | -0.03409 | 2.06E-06 | 0.000566 |
| egr1.S\|HS=EGR1\|101\|Xelaev18021326m | -1.54441 | 1.834583 | 2.60E-06 | 0.000683 |
| zfand2a.L\|HS=ZFAND2B\|100\|Xelaev18045247m | 1.492053 | 1.626419 | 3.77E-06 | 0.000926 |
| unnamed\|HS=ZNF268\|69\|Xelaev18030475m | 1.56899 | 1.525652 | 3.93E-06 | 0.000946 |
| Xetrov90011866m.L\|HS=CYP4B1\|68\|Xelaev18023138m | -1.18834 | 1.847517 | 2.07E-05 | 0.003563 |
| unnamed\|HS=NA\|00\|Xelaev18041756m | -1.12614 | 1.809542 | 2.36E-05 | 0.003877 |
| unnamed\|HS=CMKLR1\|94\|Xelaev18036272m | 1.147903 | 1.906023 | 2.62E-05 | 0.004227 |
| Xetrov90009297m.S\|HS=NA\|00\|Xelaev18020439m | -1.67421 | 0.575904 | 3.44E-05 | 0.005335 |
| unnamed\|HS=F13B\|52\|Xelaev18040622m | -2.21487 | -0.16122 | 4.44E-05 | 0.006334 |
| unnamed\|HS=ABCB1\|97\|Xelaev18030832m | 1.375761 | 1.098778 | 4.81E-05 | 0.006697 |
| fgg.S\|HS=FGG\|90\|Xelaev18009208m | -1.13076 | 3.303891 | 5.80E-05 | 0.007597 |
| syngap1.L\|HS=SYNGAP1\|98\|Xelaev18039117m | -1.56728 | 0.847605 | 5.97E-05 | 0.007776 |
| edar.L\|HS=EDAR\|97\|Xelaev18012916m | -1.29075 | 1.465492 | 7.16E-05 | 0.008778 |
| unnamed\|HS=NA\|00\|Xelaev18027435m | 1.120538 | 2.036142 | 7.19E-05 | 0.00878 |
| LOC100496812.1\|HS=TTN\|0\|Xelaev18003132m | -1.34287 | 1.283901 | 8.10E-05 | 0.009693 |
| unnamed\|HS=PHYH\|52\|Xelaev18038696m | 1.19569 | 1.748681 | 0.000108 | 0.012219 |
| emc4.S\|HS=EMC4\|71\|Xelaev18010312m | 1.02012 | 2.073805 | 0.000114 | 0.01284 |
| unnamed\|HS=PLSCR5\|69\|Xelaev18003275m | -2.24362 | -0.14107 | 0.000115 | 0.012882 |
| Xetrov90030519m.S\|HS=SULT6B1\|99\|Xelaev18029010m | 2.073631 | 0.117105 | 0.000116 | 0.012926 |
| sema3e.L\|HS=SEMA3E\|32\|Xelaev18017805m | -1.25207 | 1.378486 | 0.000117 | 0.012926 |
| unnamed\|HS=NA\|00\|Xelaev18001663m | -1.74659 | 0.318831 | 0.00012 | 0.013291 |
| apbb1ip.S\|HS=APBB1IP\|73\|Xelaev18032896m | 1.943802 | 0.043842 | 0.000131 | 0.013705 |
| ddah1.L\|HS=DDAH1\|98\|Xelaev18022880m | -1.24371 | 1.377174 | 0.000136 | 0.014151 |
| unnamed\|HS=NA\|00\|Xelaev18003792m | -1.37139 | 1.11814 | 0.000143 | 0.014606 |
| LOC100496770-like.L\|HS=FMO5\|93\|Xelaev18023617m | 1.51632 | 0.550048 | 0.000226 | 0.021005 |
| lmbrd2.S\|HS=LMBRD2\|103\|Xelaev18011016m | -1.23873 | 1.125519 | 0.000257 | 0.022898 |
| LOC100497740.S\|HS=APOH\|53\|Xelaev18037886m | -1.52138 | 0.77228 | 0.00028 | 0.024321 |
| unnamed\|HS=NA\|00\|Xelaev18026943m | 1.739503 | 0.494769 | 0.000283 | 0.024478 |
| unnamed\|HS=NA\|00\|Xelaev18019677m | 1.099027 | 1.357835 | 0.000353 | 0.029293 |
| CXCL11.L\|HS=CXCL10\|46\|Xelaev18005844m | 1.899523 | 0.320042 | 0.000354 | 0.029293 |
| unnamed\|HS=NA\|00\|Xelaev18012711m | 1.697607 | 0.18712 | 0.000398 | 0.03158 |
| unnamed\|HS=NA\|00\|Xelaev18035105m | -1.38154 | 0.986663 | 0.000441 | 0.033981 |
| cacna1f.1\|HS=CACNA1F\|40\|Xelaev18002817m | -1.32516 | 0.837765 | 0.000497 | 0.037512 |
| unnamed\|HS=PECAM1\|80\|Xelaev18046871m | -1.79216 | -0.03559 | 0.000561 | 0.040046 |
| cnih2.S\|HS=CNIH2\|86\|Xelaev18024167m | -1.00793 | 1.656352 | 0.000593 | 0.041728 |
| unnamed\|HS=KLF2\|32\|Xelaev18006666m | -1.38981 | 1.077504 | 0.000608 | 0.042504 |
| unnamed\|HS=NA\|00\|Xelaev18046811m | 1.24224 | 1.081501 | 0.000616 | 0.042888 |
| phospho1-like.S\|HS=PHOSPHO1\|81\|Xelaev18046022m | -1.15645 | 1.152413 | 0.00066 | 0.045058 |
| cyp46a1-like.2.1\|HS=CYP46A1\|95\|Xelaev18003719m | 1.445536 | 0.495885 | 0.000674 | 0.045676 |
| unnamed\|HS=NA\|00\|Xelaev18047780m | -1.96497 | -0.34768 | 0.000699 | 0.047084 |

**Supplementary Table 2. Go Term pathway analysis on differentially expressed gene by particulate matter treatment.**

| **Category name (Accession)** | **# genes** | **Percent of gene hit against total # genes** | **Percent of gene hit against total # Function hits** |
| --- | --- | --- | --- |
| **Apoptosis signaling pathway (P00006)** | **1** | **2.30%** | **3.40%** |
| **Angiogenesis (P00005)** | **1** | **2.30%** | **3.40%** |
| **Interleukin signaling pathway (P00036)** | **1** | **2.30%** | **3.40%** |
| **Alzheimer disease-amyloid secretase pathway (P00003)** | **1** | **2.30%** | **3.40%** |
| **Insulin/IGF pathway-mitogen activated protein kinase kinase/MAP kinase cascade (P00032)** | **1** | **2.30%** | **3.40%** |
| **Inflammation mediated by chemokine and cytokine signaling pathway (P00031)** | **1** | **2.30%** | **3.40%** |
| **Angiotensin II-stimulated signaling through G proteins and beta-arrestin (P05911)** | **1** | **2.30%** | **3.40%** |
| **Gonadotropin-releasing hormone receptor pathway (P06664)** | **4** | **9.10%** | **13.80%** |
| **Vitamin D metabolism and pathway (P04396)** | **1** | **2.30%** | **3.40%** |
| **PDGF signaling pathway (P00047)** | **2** | **4.50%** | **6.90%** |
| **Nicotinic acetylcholine receptor signaling pathway (P00044)** | **1** | **2.30%** | **3.40%** |
| **Oxytocin receptor mediated signaling pathway (P04391)** | **1** | **2.30%** | **3.40%** |
| **Blood coagulation (P00011)** | **3** | **6.80%** | **10.30%** |
| **B cell activation (P00010)** | **1** | **2.30%** | **3.40%** |
| **CCKR signaling map (P06959)** | **2** | **4.50%** | **6.90%** |
| **Beta2 adrenergic receptor signaling pathway (P04378)** | **1** | **2.30%** | **3.40%** |
| **Huntington disease (P00029)** | **1** | **2.30%** | **3.40%** |
| **Beta1 adrenergic receptor signaling pathway (P04377)** | **1** | **2.30%** | **3.40%** |
| **5HT2 type receptor mediated signaling pathway (P04374)** | **1** | **2.30%** | **3.40%** |
| **T cell activation (P00053)** | **1** | **2.30%** | **3.40%** |
| **Plasminogen activating cascade (P00050)** | **2** | **4.50%** | **6.90%** |
